# Using PBPK to Simulate Target Biopredictive Dissolution Profiles for Long‐Acting Injectables ‐ Where to Begin With Critical Bioavailability Attributes?

**DOI:** 10.1002/psp4.70212

**Published:** 2026-02-18

**Authors:** Hannah Cleary, Nikoletta Fotaki, Tim Persoons, Deirdre M. D'Arcy

**Affiliations:** ^1^ EPSRC‐Research Ireland Centre for Doctoral Training in Transformative Pharmaceutical Technologies, School of Pharmacy and Pharmaceutical Sciences, Trinity College Dublin Dublin Ireland; ^2^ SSPC, the Research Ireland Centre for Pharmaceuticals, School of Pharmacy and Pharmaceutical Sciences, Trinity College Dublin Dublin Ireland; ^3^ Department of Life Sciences, University of Bath Bath UK; ^4^ Department of Mechanical Manufacturing and Biomedical Engineering, Trinity College Dublin Dublin Ireland

**Keywords:** bioavailability attributes, biopredictive performance testing, dissolution testing, long‐acting injection, pharmacokinetic modeling

## Abstract

Long‐acting injectables (LAI) are of increasing interest as they facilitate improved medication adherence and exposure, with target plasma concentration levels maintained over weeks/months. Biopredictive in vitro dissolution tests can aid formulation development of LAIs and guide quality control dissolution testing by facilitating accelerated test development. However, it is not easy to develop such tests when mechanisms underlying in vivo dissolution are not fully understood. The question of interest (QOI) and context of use (COU) of this study involve quantifying the impact of in vivo parameters which are critical bioavailability attributes (CBAs), using physiologically based pharmacokinetic (PBPK) models generated for LAI methylprednisolone acetate. Simulated dissolution profiles from the PBPK models can provide a design space for biopredictive in vitro dissolution testing methods. The five CBAs explored in this study were particle size, solubility, diffusion layer thickness, diffusion coefficient, and depot volume. Although the best performing models displayed good predictive ability, they used different (literature/prediction derived) attribute values. Simulated in vivo dissolution profiles generated suggested much slower dissolution rates, with 80–100% dissolved after 1200 h, than in vitro dissolution tests from FDA ‘Dissolution Methods Database,’ where almost 90% was dissolved in 90 min. To conclude, in vitro dissolution conditions resulting in larger effective particle sizes and diffusion layer thickness, suggesting low fluid velocities, need to be explored to generate biopredictive dissolution profiles. The current approach illustrates how using models with plausible CBA value ranges can be used to simulate a target dissolution profile design space, assisting in vitro LAI dissolution test development.

## Study Highlights

**What Is the Current Knowledge on the Topic?:**

○ Long‐acting injectables (LAIs), once injected into either the muscle/adipose tissue, display an initial burst release, then a drug pocket known as a depot is formed. From this depot, drug is released into the systemic circulation over a period of time depending on physiological and formulation‐related critical bioavailability attributes.

**What Question Did This Study Address?:**

○ It is unclear to what extent/which critical bioavailability attributes affect absorption of drug from the depot. The evolving nature of dissolution methods for LAIs is reflected in the FDA ‘Dissolution Methods Database,’ highlighting the need to investigate dissolution rates which would align with observed in vivo data.

**What Does This Study Add to Our Knowledge?:**

○ This study presents simulated dissolution profiles of an LAI product correlated to an in vivo dataset providing a design space for more biorelevant dissolution tests. It also suggests parameters that have a notable impact on the biopharmaceutics aspects of the LAI PBPK model.

**How Might This Change Drug Discovery, Development, and/or Therapeutics?:**

○ This research will provide a starting point for developing more biopredictive LAI dissolution tests.

## 1 Introduction

Long acting injectables (LAIs) are beneficial due to their ability to improve patient medication adherence, especially where compliance may be an issue [1]. In a 20‐year study of anti‐psychotic patients, the use of LAIs has been associated with decreased mortality and reduced hospitalisations in comparison with outcomes from oral formulations [2].

Other medical conditions where the sustained release function of LAIs may be of benefit is in patients with human immunodeficiency virus (HIV), where there are many medications to take every day, or in dementia, where patients may simply not remember to take medications consistently [3]. Simplifying medication regimens is an advantage of LAIs over oral medications.

**FIGURE 1:**
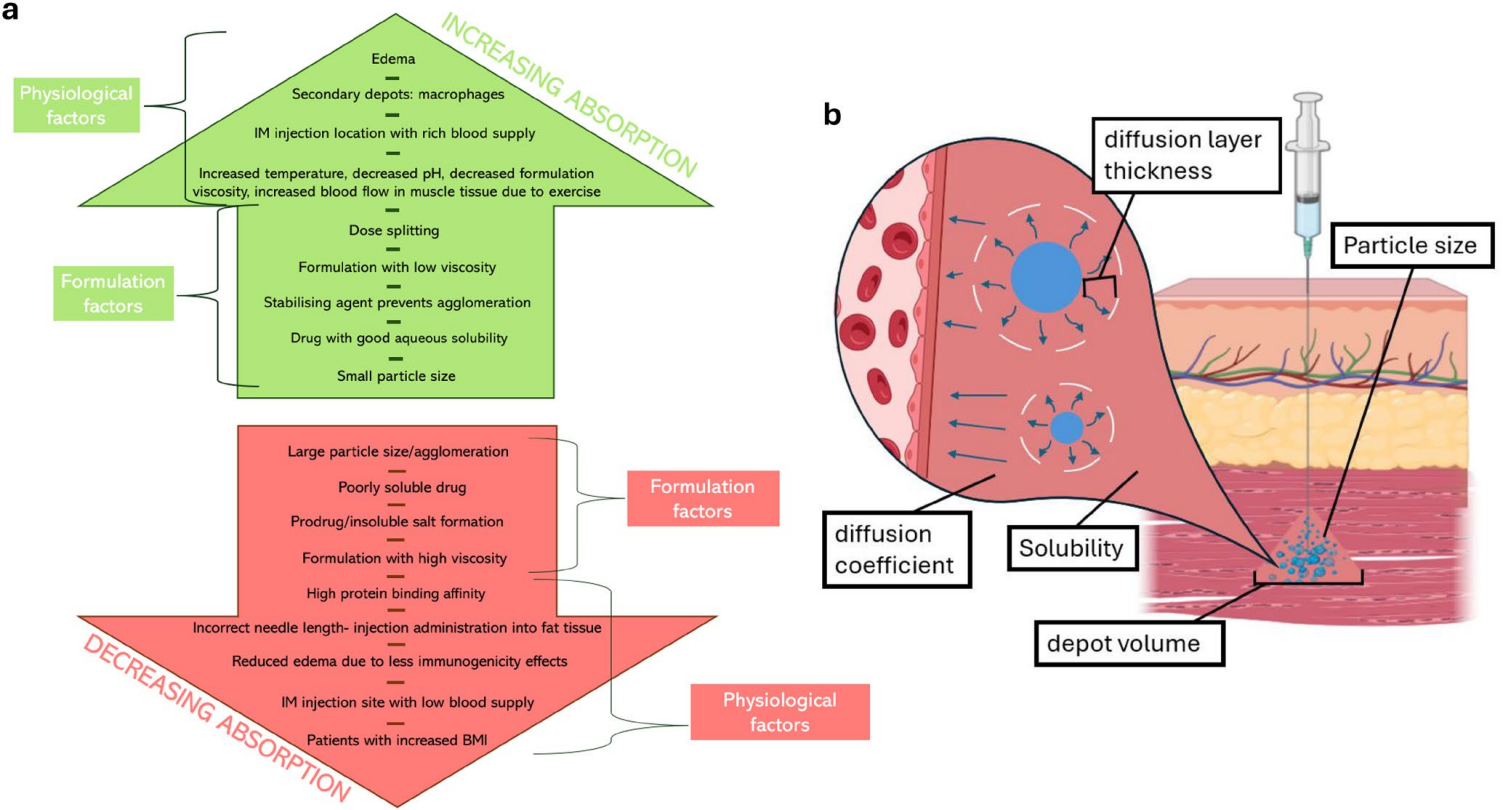
(a) A depiction of critical formulation and physiological attributes relating to long‐acting injectable preparation bioavailability. (b) The five critical bioavailability attributes explored in this work. Graphic acknoweldgement to Biorender (https://www.biorender.com/).

LAIs are usually formulated as either subcutaneous (SC) or intramuscular (IM) injections. Once injected, an initial ‘burst’ of medication is released into the blood stream as some active pharmaceutical ingredient (API) will be present in the vehicle solution when it is absorbed. Following this, a pocket of drug known as a ‘depot’ is formed between muscle/adipose cells, where drug is absorbed at a prolonged rate, dependent on critical formulation and physiological bioavailability attributes, Figure 1 [1].

Methylprednisolone acetate is a poorly soluble steroid and is available as the LAI suspension Depo‐Medrone/Depo‐Medrol, used for dermatological and rheumatic conditions, with a focus on IM administration in this study (Summary of Product Characteristics (SPC)) [4, 5].

Studies on pharmacokinetics (PK) of methylprednisolone acetate in humans are limited, with most studies examining horses and dogs [6, 7]. It can be noted that the methylprednisolone acetate injected into the muscular tissue is metabolized to methylprednisolone via butyrylcholinesterase, with methylprednisolone concentrations presented in PK studies. It is unclear if this metabolism occurs in the drug depot, in the systemic circulation, or proportions occurring at both sites [8].

The PK profile of LAIs can be complex as there are many factors at play for each stage of a drug lifecycle in the body i.e., during absorption, distribution, metabolism and excretion [9, 10]. LAIs are known to have flip‐flop kinetics, where the rate of absorption is slower than the rate of elimination [10]. Time taken to reach maximum drug concentration in plasma (*T*_max_) is generally longer in LAIs than oral formulations as more gradual absorption takes place, which is why dose loading is performed with some patients [10]. Various approaches have been used to alter the release of API for LAIs such as in situ forming gels, biodegradable microspheres and oily solutions [11]. For methylprednisolone acetate, it is the slow dissolution of poorly soluble API that provides the prolonged release effect and values of some critical aspects impacting dissolution in vivo such as effective particle size and solubility are unknown.

In PK modeling, drug dissolution in the depot prior to absorption can be simulated based on physiochemical and physiological model inputs such as solubility and particle size, or dissolution results from in vitro tests can be used as the basis for an input profile.

Dissolution/release testing is an in vitro test usually conducted in specific pharmacopeial apparatuses to measure drug dissolution/release over time. This test is not always predictive of in vivo release but is vital to guide formulation development of generic products and for quality control (QC) testing purposes, where the impact of process changes on critical quality attributes is assessed. A biopredictive test combined with PBPK modeling can be used to assess potential bioequivalence issues. As LAI PK can be complex, biopredictive dissolution/release tests need to be developed to more accurately predict in vivo data. However, without knowing values for critical bioavailability attributes (CBAs) such as solubility and effective mean particle radius (MPR) in vivo, it can be difficult to develop such tests. Establishing the impact of these attributes within reasonable boundaries enables the creation of a design space for target biopredictive dissolution profiles, thus useful to guide biopredictive test development.

There is a lack of compendial LAI dissolution test methods, but efforts are being made to explore this particularly using medroxyprogesterone acetate and paliperidone palmitate LAIs in terms of in vitro testing and convolution methods to identify even longer acting formulations than are currently available [12, 13, 14]. In particular, there is a need to investigate parameters relating to bioavailability which would align in vitro dissolution data combined with modeling studies with the observed in vivo data.

Using methylprednisolone acetate injectable suspension as a model LAI formulation, this work aims to use a PBPK modeling approach to (a) understand the magnitude of the impact of bioavailability attributes of the formulation and physiological parameters impacting bioavailability, for which in vivo attribute values are unknown, and (b) generate target dissolution profiles to determine a design space for dissolution test development.

## 2 Methods

**FIGURE 2:**
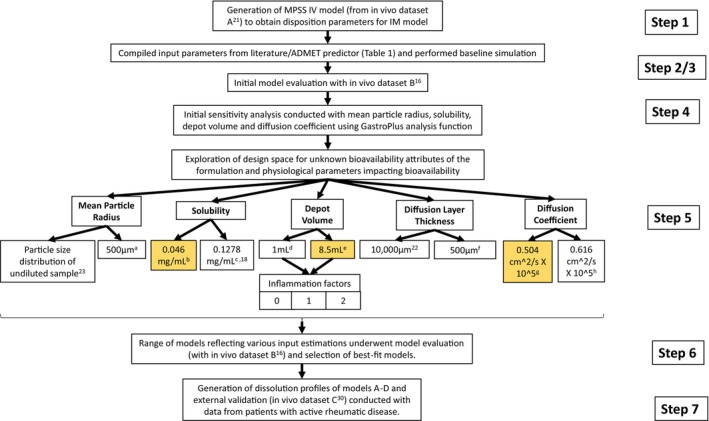
The workflow approach used for the modeling and simulation studies, guided by the EMA's ‘Guideline on the reporting of physiologically based pharmacokinetic modelling and simulation.’ [15]. Values replacing inputs in italics in Table 1. (a) rationale based on larger particles of the undiluted particle size distribution measured with a radius between 262 μm and 750 μm; (b) aqueous solubility of methylprednisolone acetate calculated by ADMET predictor; (c) average estimate of reported solubility of methylprednisolone measured in sodium phosphate buffer (PBS) containing 0.02% w/v of sodium dodecyl sulfate (SDS) and simulated healthy synovial fluid, 0.1187 and 0.1369, respectively [18]; (d) depot volume value of 1 mL was calculated based on Darville et al. [19]; (e) Default GastroPlus value based on a 1 mL injection volume; (f) diffusion layer thickness of 500 μm chosen based on mean particle radius estimation of 500 μm; (g) diffusion coefficient calculated based on the viscosity of interstitial fluid [17, 20]; (h) diffusion coefficient value of methylprednisolone acetate calculated by ADMET predictor.

GastroPlus v8.3.3 (www.simulations‐plus.com/software/gastroplus/) was used to conduct the in silico mechanistic modeling. The European Medicines Agency's (EMA's) PBPK modeling template was used as a guide as seen in workflow, Figure 2 [15].

### 2.1 Step 1 Disposition Analysis

Methylprednisolone sodium succinate (80 mg/mL) is a solution delivered intravenously (IV) not requiring in vivo dissolution and absorption; therefore, it is used to estimate disposition parameters, e.g., distribution and clearance, to be used in the IM model. In vivo data from literature was utilized (in vivo dataset A) to validate the disposition parameters (Figure S1, Table S1) [21]. Data was extracted from in vivo datasets using WebPlotDigitiser (https://automeris.io) (Supporting Information—S2 2.10.1 In vivo datasets). Through Simulations Plus' PKPlus function, 1−/2−/3‐compartment models were fit to the data, with the best model selected using model evaluation described in step 6.

### 2.2 Step 2 Formulation Inputs

Drug physicochemical properties, critical formulation attributes and physiological bioavailability attribute inputs were compiled as presented in Table 1, along with disposition parameters calculated in step 1. Where values could not be found in literature, ADMET predictor (SimulationsPlus), which uses the drug's chemical structure to estimate values related to its absorption, distribution, metabolism and excretion, was used.

**TABLE 1:** Input values gathered through a variety of sources (step 2) and used in IM methylprednisolone acetate baseline simulation (Table S4: Simulation #2). Where values could not be found in literature, ADMET predictor (SimulationsPlus), which uses the drug's chemical structure to estimate values related to its absorption, distribution, metabolism and excretion, was used. Values in italics are refined and modified in step 5. When methylprednisolone is used (due to uncertainty of methylprednisolone acetate to methylprednisolone conversion location and differences in their solubilities) in simulations, some inputs are changed as follows:to reflect methylprednisolone rather than methylprednisolone acetate: molecular weight (374.5 g), molecular formula (C22H30O3), LogP (1.525), initial dose (71.9 mg), and Peff (2.406).

Input	Value	Source
Molecular weight	416.51 g	FDA CDER chemistry review [16]
Molecular formula	C24H32O6	FDA CDER chemistry review [16]
LogP	1.467	Sandoz Canada Product Monograph ‘MPA’ Injectable Suspension USP [5]
Dosage form	IM: Suspension	Sandoz Canada Product Monograph ‘MPA’ Injectable Suspension USP [5]
Initial dose	80 mg	Sandoz Canada Product Monograph ‘MPA’ Injectable Suspension USP [5]
Dose volume	1 mL	Sandoz Canada Product Monograph ‘MPA’ Injectable Suspension USP [5]
Water solubility	*0.046 mg/mL*	ADMET predictor
Diffusion coefficient	*0.616 cm^2/s × 10^5*	ADMET predictor
Drug particle density	1.2 g/mL	GastroPlus default
Peff	2.371 cm/s × 10^4	ADMET predictor
Particle size	*25 μm*	GastroPlus default
Diffusion layer thickness	*30 μm*	GastroPlus default
Depot volume	*8.5 mL*	GastroPlus calculation
Fu tissue	37.4%	Calculated from Poulin equation using fup from SPC [4]
PK model	Compartmental (2‐compartment)	PKPlus simulation
Body weight	70 kg	GastroPlus default
FPE liver	29.2%	ADMET predictor
Fraction unbound (Fup%)	23%	SPC [4]
Volume of distribution (Vd)	0.84106 L/kg	PKPlus simulation (step 1)
Blood to plasma concentration ratio	0.799	ADMET predictor
Clearance (Cl)	0.46751 L/h/kg	PKPlus simulation (step 1)
K12	45.107 1/h	PKPlus simulation (step 1)

### 2.3 Step 3 Baseline Simulation

All inputs from Table 1 were used to generate the IM baseline simulation (Figure S2). This was compared visually and statistically with in vivo dataset B (Model Evaluation) [16].

### 2.4 Step 4 Parameter Sensitivity Analysis (PSA)

A PSA was conducted on four CBAs (Figure S3): diffusion coefficient (DC), MPR, solubility, and depot volume (DV). It was not possible to include diffusion layer thickness (DLT) with the software platform used for this PSA.

### 2.5 Step 5: Model Modification and Refining

A series of models were generated based on parameter level estimates (step 5 A‐D), Table 2. Models generated were compared visually and statistically with in vivo dataset B (Step 6: Model Evaluation) [16].

**TABLE 2:** Varying Inputs used in GastroPlus models: depot volume (DV), diffusion layer thickness (DLT), solubility, mean particle radius (MPR)/particle size distribution (PSD) and diffusion coefficient (DC).

Input parameter	Lower level	Higher level
DV (mL)	1 [19]	8.5^a^
DLT (μm)	500^b^	10,000 [22]
Solubility (mg/mL)	0.046^c^	0.1278 [18]
MPR/PSD (μm)	PSD undiluted sample [23]	500^b^
DC (cm^2/s^ × 10^5^)	0.504 [17]	0.616^c^

^a^ GastroPlus default value calculated based on injection volume of 1 mL into muscle tissue.

^b^ Estimation based on MPR estimation of 500 μm.

^c^ Value calculated through ADMET predictor.

Methylprednisolone acetate inputs (Table 1) were used with the corresponding estimated methylprednisolone acetate DC value (step 5A), whereas the estimated methylprednisolone DC value was used with methylprednisolone inputs (step 5A). Methylprednisolone inputs include those in Table 1 except: molecular weight (374.5 g), molecular formula (C22H30O3), LogP (1.525), initial dose (71.9 mg), and Peff (2.406).

### 2.6 Step 5A Solubility and DC Estimation

Two values for each of these unknown parameters were chosen based on assumptions informed by literature/predicted values but resulting in distinctly different inputs (Table 2). Solubility estimates for both methylprednisolone acetate and methylprednisolone were explored as it is uncertain to what extent the conversion of methylprednisolone acetate to methylprednisolone by butyrylcholinesterase occurs in the drug depot and in the systemic circulation or a proportion in both [8].

The methylprednisolone solubility value was an average estimate of reported solubility of methylprednisolone measured in sodium phosphate buffer (PBS) containing 0.02% w/v of sodium dodecyl sulfate (SDS) and simulated healthy synovial fluid, 0.1187 mg/mL and 0.1369 mg/mL, respectively [18]. Methylprednisolone acetate solubility was the predicted aqueous solubility from ADMET Predictor, representing two potential extremes of solubility.

The higher DC value chosen was based on the aqueous prediction (ADMET Predictor) for methylprednisolone acetate, and the lower value was calculated based on the viscosity of interstitial fluid as presented by Torres‐Teran et al. [17]. The calculation was based on the Stokes‐Einstein relation D∝1/ƞ, where D is DC and ƞ is viscosity [20].

### 2.7 Step 5B MPR/PSD Estimation

Two approaches were used for particle size inputs‐ a PSD and MPR estimation. Undiluted PSD data was used from a study by Benzon et al. where 40 mg/mL Depo‐Medrol injections under a Zeiss LSM 510 laser scanning confocal microscope, were measured and analyzed with velocity software, Improvision Openlab 3.1.1, Table S2 [23].

As an alternative input, an MPR value of 500 μm was selected based on the rationale that particle agglomeration is likely in the depot site, and the larger particles of the undiluted PSD were observed to have a radius between 262 μm and 750 μm [23].

### 2.8 Step 5C DLT Estimation

Values at two levels for DLT were also examined. One level had a DLT value of 1 cm (10,000 μm), previously employed in the modeling of cabotegravir by Lukacova et al. [22]. Based on its prior use in an LAI model, this high DLT value was chosen as the largest DLT value explored in our model.

Additionally, the other DLT value estimation was 500 μm, based on fluid dynamics theory which states the DLT of a dissolving particle should not exceed its radius, the estimated MPR value from step 5B [24].

### 2.9 Step 5D DV Estimation

A DV value of 8.5 mL was used as calculated by GastroPlus based on IM administration of a 1 mL injection. An additional DV value of 1 mL was used in this work, a calculated scaled value based on the work of Darville et al. who proposed DV may be less than that initially calculated due to immediate absorption of the aqueous vehicle into surrounding tissues, supported by image analysis of IM administration site micrographs examining depot geometry [19, 25]. A proportional decrease based on this study was used to calculate the 1 mL DV value.

Creating an inflammation profile: A previously reported approach involved measuring the DV change of cabotegravir LAI, where the proportional change in physical volume, compared to the initial volume post administration based on observed proportional changes measured in rats, was used [25, 26, 27, 28]. The inflammation vs. time profile, as % depot volume change over time, from this study was applied to the model as a support file for both DV values of 8.5 mL and 1 mL [27].

Inclusion of inflammation factor (IF): IF is a scaling factor of the inflammation profile. For both DV levels (8.5 mL and 1 mL), the effect of IF was examined comparing the effects of no IF and IF values of 1 and 2.

### 2.10 Step 6 Model Evaluation and Selection

**FIGURE 3:**
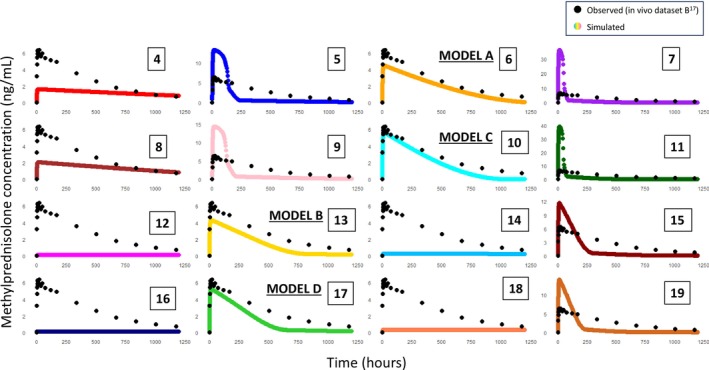
A series of simulations conducted using baseline model inputs from Table 1 and alterations as listed in Table 2, with observed data from in vivo dataset B in black and simulated data in color [16]. Simulations 4–7 and 12–15 have methylprednisolone acetate inputs, whereas simulations 8–11 and 16–19 have methylprednisolone inputs. Alternating simulations use MPR 500 μm [simulation #4, 6, 8, 10, 12 14, 18, 21] and undiluted PSD [simulation #5, 7, 9, 11, 13, 15, 17]. All simulations in columns 1 and 2 use low solubility value of 0.046 mg/mL, whereas columns 3 and 4 use the higher solubility value of 0.1278 mg/mL. Simulations 4–11 have a DLT of 500 μm, whereas simulations 12–19 have a 1 cm DLT value. Simulations 6, 13, 10 and 17 were considered acceptable during model evaluation, relabelled A, C, B, D respectively and used in subsequent steps. (Table S4: Simulations #4–19).

A range of simulations from step 5 were selected to be assessed for predictive ability (Step 6: Model Evaluation). The most predictive models were brought forward to the next steps (Figure 3).

#### 2.10.1 Model Evaluation

Accuracy of simulated profiles was assessed through comparison with observed data through visual assessment initially.

IV model: Akaike's Information Criterion (AIC), Schwarz Information Criterion (SIC), and residuals were used to assess the model, with the final selected model being the simplest model adequately describing the data.

IM models: if the predicted in vivo *C*_max_ was within approximately 2–3 fold of the observed *C*_max_, the models were selected for further evaluation using average fold error (AFE) and absolute average fold error (AAFE) on the concentration‐time data points and fold error (FE) on pharmacokinetic parameters as calculated below:

(1) $$\mathrm{AFE}=10^{\frac{1}{n}*\sum \log\left(\left|\frac{{\text{predicted}}_{i}}{{\text{observed}}_{i}}\right|\right)}.$$

(2) $$\text{AAFE}=10^{\frac{1}{n}*\sum |\log\left(\frac{{\text{predicted}}_{i}}{{\text{observed}}_{i}}\right)|}.$$

(3) $$\mathrm{FE}=\frac{{\text{predicted}}_{i}}{{\text{observed}}_{i}}.$$

where *n* is the number of time points used, predicted_i_ is the simulated concentration value at time i, and observed_i_ is the concentration value of the observed data, from in vivo dataset B, at time i. If a simulation has statistical values (e.g., FE, AAFE, AFE) between 0.5 and 2, it can be considered a valid, successful model [29].

#### 2.10.2 Step 7 Dissolution Profile Generation and External Validation

Models A‐D, based on model evaluation, were chosen for further exploration.

As part of the simulated absorption process, the PBPK model includes a simulated in vivo dissolution step and simulated absorption step. Dissolution profiles from the best performing models (A‐D) were generated based on the output simulated profile ‘amount of API released in vivo over time’ generated.

An external validation was also conducted using in vivo dataset C from literature [30], Supporting Information S2 in vivo datasets, Table S2_3. This dataset included a 120 mg/3 mL dose of methylprednisolone acetate administered on day 0 and day 15 to 48 patients with active rheumatoid arthritis (RA).

An external validation using RA patient data, incorporating patient response variability, was used to assess model C over multiple doses. Simulated plasma concentration versus time data was assessed for predictive ability (Step 6: Model Evaluation).

## 3 Results

### 3.1 Step 1 Disposition Analysis

The validation IV model of methylprednisolone sodium succinate used to generate disposition parameters for use in IM models is presented in Figure S1 (Table S4: *simulation* #1). A 2‐compartmental model was considered adequate due to the low AIC value of −89.56, very low residuals, and acceptable visual fit.

Statistical analysis (FE, AFE and AAFE), AIC, SIC and visual fit suggest the model is adequate for use to calculate disposition parameters: Figure S1, Table S1.

### 3.2 Step 2 & 3 Formulation Inputs & Baseline Simulation

The initial baseline IM simulation based on Table 1 inputs is presented in Figure S2 (Table S4: simulation #2). In this model, the *C*_max_ is far greater in the simulated (107.78 ng/mL) than in the observed data from in vivo dataset A (6.35 ng/mL). The sharp peak indicates a large amount of drug is absorbed quickly, which is not the case based on observed data. The simulated *T*_max_ (9.9 h) is also less than the observed *T*_max_ (24 h), although due to the suggested presence of multiple peaks in the in vivo concentration‐time profile, the true *T*_max_ could be any point from a range of peaks close in value to the early (< 24 h) timepoints. Overall, the observed data is not well represented by this model.

### 3.3 Step 4 Initial PSA

The PSA results, in Figure S3, suggest that MPR has a notable effect on *C*_max_ concentrations, *T*_max_ and AUC_0‐t_ for the range investigated (Table S4: simulation #3). Although solubility and DV were seen to impact *C*_max_ and *T*_max_, the suggested impact of DC was less within the range explored.

### 3.4 Step 5: Model Modification and Refining

A series of models were created based on refining the baseline model, outlined in Step 5 A‐D, as presented in Figure 3. Using methylprednisolone acetate inputs rather than methylprednisolone inputs did not have a notable effect on simulation results. For example, simulated profiles 4–7 with methylprednisolone acetate inputs versus simulated profiles 8–11 with methylprednisolone inputs have the same shape with slightly lower *C*_max_ values in methylprednisolone input simulated data, Figure 3.

### 3.5 Step 5A Solubility and DC Estimation

A higher solubility value increased the peak plasma concentration in simulations, whereas DC did not appear to have a notable impact on predicted profiles, in line with PSA results, Figure 3.

### 3.6 Step 5B and C, MPR/PSD and DLT Estimation

Most simulations using undiluted PSD in Figure 3 (simulations #5, 7, 9, 11, 15, 19) were similar in shape to the baseline simulation (Figure S2) with a sharp peak initially. This is due to a significant number of particles measured being less than 10 μm in diameter. Although in vivo some particles may be this size, the effective in vivo particle size would potentially be larger, with agglomeration of particles due to the mass injected (80 mg/mL) into the available DV. The exceptions were simulations #13 and 17, which also included a combination of low solubility and a DLT of 1 cm, likely counteracting the effect of the smaller particle size on dissolution. Conversely, simulations with a single MPR of 500 μm had very low *C*_max_ values, apart from simulations #6 and 10. Both of these models included a combination of the higher solubility and lower DLT values, facilitating faster dissolution and consequently a higher *C*_max_. Overall, it is clear from Figure 3 that a DLT of 1 cm is more predictive of in vivo data when combined with the undiluted PSD data, and a DLT of 500 μm is more predictive when combined with the MPR of 500 μm.

### 3.7 Step 5D DV Estimation

The greatest impact from changing DV from 8.5 mL to 1 mL was with *C*_max_ with approximately a 10% reduction. The effect of IF was also examined. Under conditions where 1 mL DV, undiluted PSD, DLT 10,000 μm, 0.1278 mg/mL solubility was used, there was approximately a 22% increase in *C*_max_ from IF0 (11.6 ng/mL) to IF1 (14.20 ng/mL) and a 4% increase in *C*_max_ from IF1 (14.20 ng/mL) to IF2 (14.79 ng/mL). This pattern is similar among all simulation conditions (Table S3).

Given the minimal impact of DV, since the injection product volume was 1 mL in an aqueous buffer allowing for absorption of some vehicle once injected, the DV of 1 mL was considered acceptable for use in all further simulations. As IF seemed to have a marginal effect on *C*_max_ but even less of an effect on *T*_max_, AUC_0‐inf_ and AUC_0‐t_, no inflammation factor was used to avoid adding additional unnecessary complexity to the model.

### 3.8 Step 6 Model Evaluation and Selection

Four models with model evaluation parameters within 2‐fold of observed data were selected as adequately predicting in vivo dataset B: (Table S4 simulation #6, 13, 10, 17, renamed A‐D respectively) [16]. These models all used different but plausible combinations of solubility, DLT, MPR/PSD values and methylprednisolone/methylprednisolone acetate specific input values, illustrating the relevance of identifying rational values for these CBAs.

### 3.9 Step 7 Dissolution Profile Generation and External Validation

**FIGURE 4:**
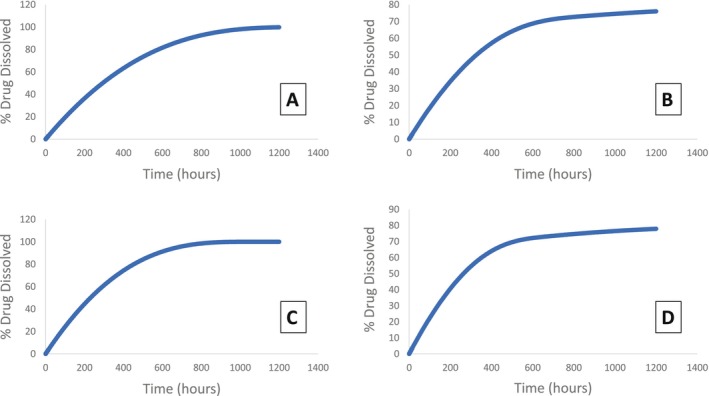
Target dissolution profiles (% drug dissolved versus time) based on simulated profiles. Target profiles from model A and C both reach 100% drug dissolved within the 1200 h time period. These models have solubility of 0.1278 mg/mL, DLT of 500 μm, and MPR of 500 μm values in common, but model A has methylprednisolone acetate inputs whereas model C has methylprednisolone inputs. In contrast, models B and D only reach approximately 80% drug dissolved within the 1200 h time period. These models have solubility of 0.046 mg/mL, DLT of 1 cm, and PSD undiluted sample values in common (Table S4: Simulations #6, 10, 13, 17).

Based on models selected in step 6, target dissolution profiles were generated (Figure 4), based on simulated in vivo dissolution (Table S4: simulations #6, 13, 10, 17, dissolution models A, B, C, and D respectively). Although these four models can adequately predict in vivo dataset B, their target dissolution profiles are not identical. Model A and C are similar in the fact that they both reach/almost reach 100% drug dissolved within the 1200 h time frame of the test, whereas model B and D reach approximately 80% dissolved after this same time frame. Models B and D have similarities—they use the same PSD, 1 cm DLT and low solubility, whereas models A and C use the MPR of 500 μm, 500 μm DLT and higher solubility value. These target dissolution profiles provide a starting point to guide development of biopredictive dissolution studies for this product.

**FIGURE 5:**
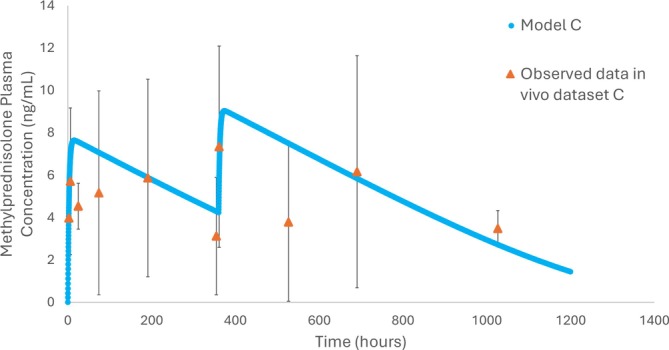
External validation of simulated plasma methylprednisolone (ng/mL) versus time (hours) of model C (blue) and in vivo dataset C (orange) [30]. This dataset assesses model C against patient concentration data over 1200 h in patients who have active rheumatoid arthritis. The dose of methylprednisolone acetate injected is 120 mg/3 mL at time zero and repeated at day 15 (360 h). AFE and AAFE were 1.17, 1.30 respectively, and FE values of *C*_max_, *T*_max_, AUC_0‐inf_ and AUC_0‐t_ were 0.81, 0.96, 1.07 and 0.8 respectively. All model evaluation assessments were acceptable (Table S4: Simulation #40).

An external validation was also conducted with in vivo dataset C with model C, Figure 5 (Table S4: simulation #40, [30]). Model C was selected over models B and D as the larger particle size and higher solubility are considered likely to be closer to in vivo values than the original undiluted PSD and predicted aqueous solubility values used in models B and D. Furthermore, model C uses methylprednisolone inputs, assuming conversion from methylprednisolone acetate in the depot site, which model A does not assume. AFE and AAFE were 1.17, 1.30 respectively, and FE values of *C*_max_, *T*_max_, AUC_0‐inf_, and AUC_0‐t_ were 0.81, 0.96, 1.07, and 0.8 respectively. Based on model evaluation, model C predicts observed dataset C with sufficient accuracy [30] (Figure 5). This external validation, based on data from patients with RA, further supports the use of this model to guide clinically relevant dissolution profile generation.

## 4 Discussion

An understanding of the impact of CBAs, MPR, and DLT in particular on PBPK model predictions will aid development of biopredictive in vitro dissolution test conditions.

As expected, particle size significantly influenced the simulated concentration‐ both in terms of C_max_ and shape. Changing one CBA e.g., MPR and not the other e.g., DLT affects the model, but together, they have a greater impact (Figure 3), illustrating the value of comprehensive and systematic in silico investigation in model development. Estimating exact in vivo particle size is challenging and it was interesting to observe simulations with measured PSDs applied. A high proportion of smaller particles were present and therefore dissolved quickly, whereas the effective in vivo PSD will likely have a smaller proportion of smaller particles and more larger particles present due to reduced space for particle movement and reduced vehicle available for dissolution leading to increased particle agglomeration. The potential for particulate dispersal to be limited to a confined depot volume following IM injection is evident in images from histological micrograph data presented by Darville et al. which characterized depot geometries following administration of paliperidone palmitate to rats [19]. Similarly, DLT is another CBA, although difficult to measure/estimate the effective thickness of the slow‐moving layer of fluid surrounding the particles in vivo.

In the current work, the large DLT was only required when the measured PSD was used. When a larger particle size was assumed (still within the measured range), a more realistic DLT resulted in reasonable simulated concentrations. The impact of methylprednisolone vs. methylprednisolone acetate inputs was minimal, but solubility did have an impact; therefore the lower predicted aqueous solubility value of methylprednisolone acetate might be more relevant at the point of dissolution, but conversion to methylprednisolone could take place prior to absorption. On the other hand, as the local environment will impact the solubility, this illustrates how critical in vivo solubility estimates are to generating relevant PBPK models. Furthermore, limitations to this study include that the true solubility may lie outside the estimated range and that datasets A, B only displayed mean observed data without variability, while dataset C did include variability in the form of standard deviation error bars.

DV and IF results suggest a minor impact on PK parameters (Table S3). Although there are studies investigating the DV, it can be difficult to capture this in vivo [19, 25, 26, 27, 28].

In terms of the implications of these CBAs on in vitro testing, it is likely that the effective in vivo particles are larger than the undiluted PSD with a DLT of similar size as particle radius, indicating a low velocity environment is required for in vitro dissolution testing conditions. A small available volume for dissolution to occur also enables settling and agglomeration of particles in vivo. Solubility is another unknown attribute as the composition of relevant IM fluid is not fully established. Although the IM environment has not been studied in terms of composition, the SC environment viscosity has been found to be between 1.0 and 1.4 mPa·s, and total protein content of interstitial fluid samples ranged from 22.0 to 57.2 g/L for all preclinical animal species studied [17]. This could be used as a basis for IM medium composition.

The question of interest (QOI) and context of use (COU) of this study involves the creation of a design space for unknown in vivo parameters (the CBAs) for the LAI to be used as a starting point for developing biopredictive in vitro dissolution testing methods [31, 32]. As outlined in the PBBM reporting template, PBBM/PBPK models can have different risk levels depending on COU and decision consequence [31, 33]. Based on this assessment, the model outlined in this study can be used at low risk with available clinical data to guide target dissolution profile generation and identify the likely ranges of values of unknown parameters and the sensitivity of the PK profile to these parameters.

The FDA's ‘Dissolution Methods Database’ lists previously used dissolution methods but for many LAIs it states, ‘develop a method to characterize in vitro release.’ A QC test needs to be appropriately discriminatory, an aspect which is particularly pertinent when accelerated test conditions are used as can be the case with LAIs due to the prolonged real‐time dissolution/release timescales. Furthermore QC dissolution testing will ideally support in vitro‐in vivo relationships. The FDA database for methylprednisolone acetate details a medium of water with 0.55% w/v sodium dodecyl sulfate, in the flow‐through dissolution apparatus at 8 mL/min for 120 min [34]. Use of these conditions has resulted in approximately 90% dissolved in 90 min [16]. The difference in timescale between this suggested test and the target dissolution profiles in the current work illustrate the need for QC tests to be both discriminatory and in vivo relevant, and the lack of understanding of certain important in vivo parameters relating to the dissolution of LAI suspensions highlights the need to explore more discriminative LAI dissolution methods. Although models A‐D used different solubilities and particle size inputs, resulting in different simulated in vivo dissolution profiles, the range of profiles generated enables identification of a target range for biopredictive dissolution profiles. Better knowledge of in vivo solubility would facilitate conditions where effective in vivo particle size could be present during in vitro dissolution. The current study suggests that particle sizes of approximately 500 μm or smaller and DLT of similar size would be a useful starting point in developing in vitro dissolution tests for methylprednisolone acetate IM suspension injections.

## Author Contributions

Deirdre M. D'Arcy, Hannah Cleary, and Nikoletta Fotaki wrote the manuscript; Deirdre M. D'Arcy, Nikoletta Fotaki, and Tim Persoons designed the research; Hannah Cleary performed the research; Hannah Cleary and Deirdre M. D'Arcy analyzed the data.

## Funding

This work was supported by Science Foundation Ireland (now Research Ireland) grant 18/EPSRC‐CDT/3587 and the Engineering and Physical Sciences Research Council EP/S023054/1.

## Conflicts of Interest

The authors declare no conflicts of interest.

## Supporting information


**Data S1:** Supporting Information.

**Data S2:** Supporting Information.
