# Supplementary material for: Using PBPK to Simulate Target Biopredictive Dissolution Profiles for Long‐Acting Injectables ‐ Where to Begin With Critical Bioavailability Attributes?
Source: CPT Pharmacometrics Syst Pharmacol. 2026 Feb 18;15(3):e70212. doi: 10.1002/psp4.70212 (PMC12916861; doi:10.1002/psp4.70212)
Supplement: Supplementary file 1 — Data S1: Supporting Information. [file PSP4-15-e70212-s002.docx]

Supplementary Information

**Step 1:**


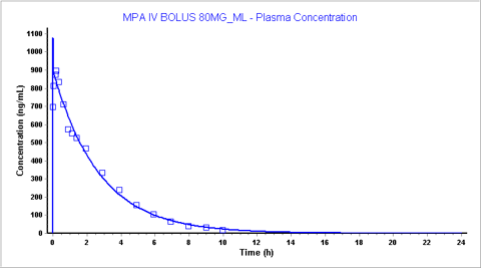

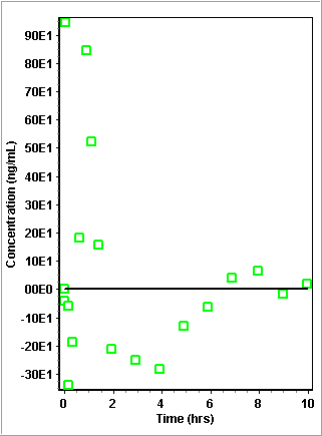


Figure S1. Concentration (ng/mL) versus time (hours) of simulated IV methylprednisolone sodium succinate (blue line) and methylprednisolone sodium succinate IV data from in vivo dataset A (blue squares) [left plot] (21). Plot of residuals for 2-compartmental model, generated by PKPlus, Simulations Plus [right plot] (*Table S4: simulation #1*).

Table S1. FE, AFE and AAFE statistical analysis of simulated MPSS values obtained for PK parameters. Simulated T_max_ was earlier than 0.18 hours with corresponding higher C_max_ but there was no corresponding data point available in in vivo dataset A, therefore 0.18 hours was used here for comparison purposes (*Table S4: simulation #1*) (21).

**Step 3:**


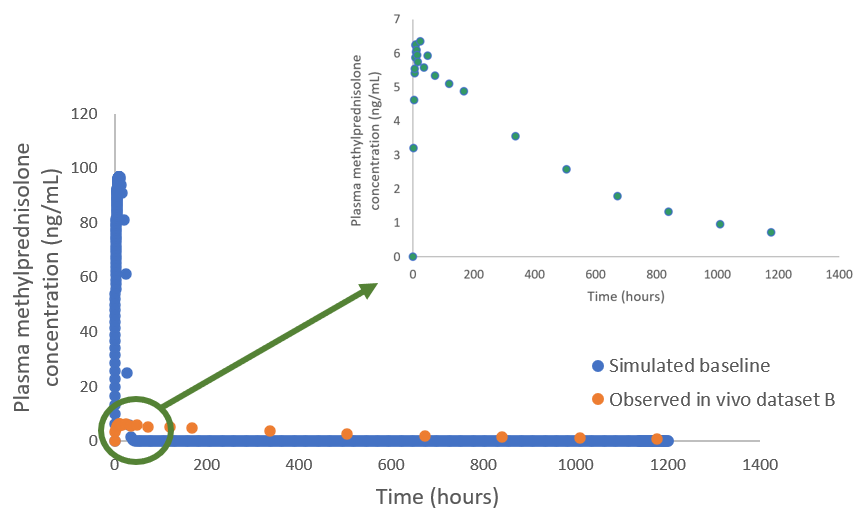


Figure S2. Plasma concentration (ng/mL) versus time (hours) of simulated methylprednisolone IM (blue) and observed methylprednisolone IM data from in vivo dataset B (orange) with inputs from Table 1. Inset focus on in vivo dataset B (*Table S4: simulation #2*) (16).

**Step 4:**


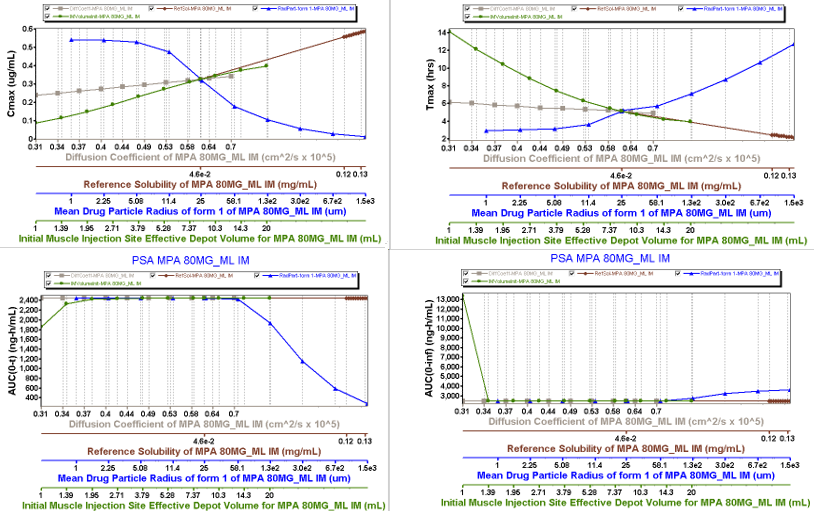


Figure S3. PK results (C_max_, T_max_, AUC_(0-t)_ and AUC_(0-inf)_) for PSA of four critical formulation and physiological bioavailability attributes: diffusion coefficient (grey), solubility (brown), MPR (blue), depot volume (green) (*Table S4: simulation #3*).

**Step 5:**

|  | Diameter (µm) | Radius (µm) | % |
| --- | --- | --- | --- |
| Undiluted | 10 | 5 | 63 |
|  | 35 | 17.5 | 14 |
|  | 525 | 262.5 | 17 |
|  | 1500 | 750 | 6 |

Table S2. Mean particle size distributions, from literature, in 80mg/mL methylprednisolone acetate Depo-Medrol injections under a Zeiss LSM 510 laser scanning confocal microscope, measured and analysed with velocity software, Improvision Openlab 3.1.1. used in step 5A (23).

**Step 5D:**

Table S3. Simulated PK parameters versus depot volume of 8.5mL (IF 0, 1, 2) and depot volume 1mL (IF 0,1, 2) compared with observed in vivo data from dataset B (*Table S4: simulations #4-7 and 20-39*) (16).

| # | Description | Inputs for methylprednisolone acetate or methylprednisolone? | Solubility (mg/mL) | DC | MPR/ PSD (µm) | DV (mL)/IF | DLT (µm) | Outcome/C_max_ (ng/mL) |
| --- | --- | --- | --- | --- | --- | --- | --- | --- |
| 1 | Step 1: Disposition analysis | Methylprednisolone sodium succinate for intravenous administration | N/A | 0.616 | N/A | N/A | N/A | Disposition parameters added to *Table1.* |
| 2 | Step 3: Baseline simulation | Methylprednisolone acetate | 0.046 | 0.616 | 25 MPR | 8.5/IF 0 | 30 | C_max_: 96.91.  C_max_ FE: 15.26.  Not within evaluation limits. |
| 3 | Step 4: Initial PSA | Methylprednisolone acetate | 0.1187-0.1369 | 0.308-0.7 | 1-1500 | 1-20/IF 0 | N/A | Further exploration required in step 5. |
| 4 | Step 5: Model modification and refining | Methylprednisolone acetate | 0.046 | 0.504 | 500 MPR | 1/IF 0 | 500 | C_max_: 1.62.  C_max_ FE: 0.26.  Not within evaluation limits. |
| 5 | Step 5: Model modification and refining | Methylprednisolone acetate | 0.046 | 0.504 | PSD undiluted sample | 1/IF 0 | 500 | C_max_: 13.25.  C_max_ FE: 2.09.  Not within evaluation limits. |
| 6 | Step 5: Model modification and refining | Methylprednisolone acetate | 0.1278 | 0.504 | 500 MPR | 1/IF 0 | 500 | C_max_: 4.46(FE 0.70).  T_max_: 15.8 (FE 0.66).  AUC_0-inf_: 2199.6 (FE 0.63).  AUC_0-t_: 2194.6 (FE 0.71).  AFE: 0.66.  AAFE: 1.39.  Within evaluation limits. Model A in step 7. |
| 7 | Step 5: Model modification and refining | Methylprednisolone acetate | 0.1278 | 0.504 | PSD undiluted sample | 1/IF 0 | 500 | C_max_: 36.16.  C_max_ FE: 5.70.  Not within evaluation limits. |
| 8 | Step 5: Model modification and refining | Methylprednisolone | 0.046 | 0.616 | 500 MPR | 1/IF 0 | 500 | C_max_: 2.05.  C_max_ FE: 0.32.  Not within evaluation limits. |
| 9 | Step 5: Model modification and refining | Methylprednisolone | 0.046 | 0.616 | PSD undiluted sample | 1/IF 0 | 500 | C_max_: 14.35.  C_max_ FE: 2.26.  Not within evaluation limits. |
| 10 | Step 5: Model modification and refining | Methylprednisolone | 0.1278 | 0.616 | 500 MPR | 1/IF 0 | 500 | C_max_: 5.62(FE 0.89).  T_max_: 15.2 (FE 0.63).  AUC_0-inf_: 2196.9 (FE 0.63).  AUC_0-t_: 2196.9 (FE 0.71).  AFE: 0.39.  AAFE: 2.57.  Within evaluation limits. Model C in step 7. |
| 11 | Step 5: Model modification and refining | Methylprednisolone | 0.1278 | 0.616 | PSD undiluted sample | 1/IF 0 | 500 | C_max_: 39.19.  C_max_ FE: 6.17.  Not within evaluation limits. |
| 12 | Step 5: Model modification and refining | Methylprednisolone acetate | 0.046 | 0.504 | 500 MPR | 1/IF 0 | 10,000 | C_max_: 0.09.  C_max_ FE: 0.01.  Not within evaluation limits. |
| 13 | Step 5: Model modification and refining | Methylprednisolone acetate | 0.046 | 0.504 | PSD undiluted sample | 1/IF 0 | 10,000 | C_max_: 4.29 (FE 0.68).  T_max_: 15.5 (FE 0.65).  AUC_0-inf_: 1807.3 (FE 0.52).  AUC_0-t_: 1671.1 (FE 0.54).  AFE: 0.56.  AAFE: 1.79.  Within evaluation limits. Model B in step 7. |
| 14 | Step 5: Model modification and refining | Methylprednisolone acetate | 0.1278 | 0.504 | 500 MPR | 1/IF 0 | 10,000 | C_max_: 0.25.  C_max_ FE: 0.04.  Not within evaluation limits. |
| 15 | Step 5: Model modification and refining | Methylprednisolone acetate | 0.1278 | 0.504 | PSD undiluted sample | 1/IF 0 | 10,000 | C_max_: 11.61.  C_max_ FE: 1.83.  Not within evaluation limits. |
| 16 | Step 5: Model modification and refining | Methylprednisolone | 0.046 | 0.616 | 500 MPR | 1/IF 0 | 10,000 | C_max_: 0.12.  C_max_ FE: 0.02.  Not within evaluation limits |
| 17 | Step 5: Model modification and refining | Methylprednisolone | 0.046 | 0.616 | PSD undiluted sample | 1/IF 0 | 10,000 | C_max_: 5.21 (FE 0.82).  T_max_: 15.1 (FE 0.63).  AUC_0-inf_: 1791.7 (FE 0.51).  AUC_0-t_: 1711.1 (FE 0.55).  AFE: 0.57.  AAFE: 1.75.  Within evaluation limits. Model D in step 7. |
| 18 | Step 5: Model modification and refining | Methylprednisolone | 0.1278 | 0.616 | 500 MPR | 1/IF 0 | 10,000 | C_max_: 0.33.  C_max_ FE: 0.05.  Not within evaluation limits. |
| 19 | Step 5: Model modification and refining | Methylprednisolone | 0.1278 | 0.616 | PSD undiluted sample | 1/IF 0 | 10,000 | C_max_: 14.05.  C_max_ FE: 2.21.  Not within evaluation limits. |
| 20 | Step 5D: DV Model modification and refining | Methylprednisolone acetate | 0.046 | 0.504 | 500 MPR | 8.5/IF 0 | 500 | C_max_: 1.79.  C_max_ FE: 0.28.  Not within evaluation limits. |
| 21 | Step 5D: DV Model modification and refining | Methylprednisolone acetate | 0.046 | 0.504 | 500 MPR | 8.5/IF 1 | 500 | C_max_: 1.81.  C_max_ FE: 0.29.  Not within evaluation limits. |
| 22 | Step 5D: DV Model modification and refining | Methylprednisolone acetate | 0.046 | 0.504 | 500 MPR | 8.5/IF 2 | 500 | C_max_: 1.81.  C_max_ FE: 0.29.  Not within evaluation limits. |
| 23 | Step 5D: DV Model modification and refining | Methylprednisolone acetate | 0.1278 | 0.504 | 500 MPR | 8.5/IF 0 | 500 | C_max_: 4.92.  C_max_ FE: 0.77.  Within evaluation limits for C_max_. |
| 24 | Step 5D: DV Model modification and refining | Methylprednisolone acetate | 0.1278 | 0.504 | 500 MPR | 8.5/IF 1 | 500 | C_max_: 4.98.  C_max_ FE: 0.78.  Within evaluation limits for C_max_. |
| 25 | Step 5D: DV Model modification and refining | Methylprednisolone acetate | 0.1278 | 0.504 | 500 MPR | 8.5/IF 2 | 500 | C_max_: 4.98.  C_max_ FE: 0.78.  Within evaluation limits for C_max_. |
| 26 | Step 5D: DV Model modification and refining | Methylprednisolone acetate | 0.046 | 0.504 | PSD undiluted sample | 8.5/IF 0 | 10,000 | C_max_: 5.70.  C_max_ FE: 0.90.  Within evaluation limits for C_max_. |
| 27 | Step 5D: DV Model modification and refining | Methylprednisolone acetate | 0.046 | 0.504 | PSD undiluted sample | 8.5/IF 1 | 10,000 | C_max_: 5.94.  C_max_ FE: 0.94.  Within evaluation limits for C_max_. |
| 28 | Step 5D: DV Model modification and refining | Methylprednisolone acetate | 0.046 | 0.504 | PSD undiluted sample | 8.5/IF 2 | 10,000 | C_max_: 5.95.  C_max_ FE: 0.94.  Within evaluation limits for C_max_. |
| 29 | Step 5D: DV Model modification and refining | Methylprednisolone acetate | 0.1278 | 0.504 | PSD undiluted sample | 8.5/IF 0 | 10,000 | C_max_: 15.20.  C_max_ FE: 2.39.  Not within evaluation limits. |
| 30 | Step 5D: DV Model modification and refining | Methylprednisolone acetate | 0.1278 | 0.504 | PSD undiluted sample | 8.5/IF 1 | 10,000 | C_max_: 15.80.  C_max_ FE: 2.49.  Not within evaluation limits. |
| 31 | Step 5D: DV Model modification and refining | Methylprednisolone acetate | 0.1278 | 0.504 | PSD undiluted sample | 8.5/IF 2 | 10,000 | C_max_: 15.81.  C_max_ FE: 2.49.  Not within evaluation limits. |
| 32 | Step 5D: DV Model modification and refining | Methylprednisolone acetate | 0.046 | 0.504 | 500 MPR | 1/IF 1 | 500 | C_max_: 1.75.  C_max_ FE: 0.28.  Not within evaluation limits. |
| 33 | Step 5D: DV Model modification and refining | Methylprednisolone acetate | 0.046 | 0.504 | 500 MPR | 1/IF 2 | 500 | C_max_: 1.78.  C_max_ FE: 0.28.  Not within evaluation limits. |
| 34 | Step 5D: DV Model modification and refining | Methylprednisolone acetate | 0.1278 | 0.504 | 500 MPR | 1/IF 1 | 500 | C_max_: 4.83.  C_max_ FE: 0.76.  Within evaluation limits for C_max_. |
| 35 | Step 5D: DV Model modification and refining | Methylprednisolone acetate | 0.1278 | 0.504 | 500 MPR | 1/IF 2 | 500 | C_max_: 4.89.  C_max_ FE: 0.77.  Within evaluation limits for C_max_. |
| 36 | Step 5D: DV Model modification and refining | Methylprednisolone acetate | 0.046 | 0.504 | PSD undiluted sample | 1/IF 1 | 10,000 | C_max_: 5.23.  C_max_ FE: 0.82.  Within evaluation limits for C_max_. |
| 37 | Step 5D: DV Model modification and refining | Methylprednisolone acetate | 0.046 | 0.504 | PSD undiluted sample | 1/IF 2 | 10,000 | C_max_: 5.48.  C_max_ FE: 0.86.  Within evaluation limits for C_max_. |
| 38 | Step 5D: DV Model modification and refining | Methylprednisolone acetate | 0.1278 | 0.504 | PSD undiluted sample | 1/IF 1 | 10,000 | C_max_: 14.20.  C_max_ FE: 2.24.  Not within evaluation limits. |
| 39 | Step 5D: DV Model modification and refining | Methylprednisolone acetate | 0.1278 | 0.504 | PSD undiluted sample | 1/IF 2 | 10,000 | C_max_: 14.79.  C_max_ FE: 2.33.  Not within evaluation limits. |
| 40 | Step 7 External validation: model C | Methylprednisolone inputs with dose change to 120mg/3ml on day 0 and day 15 | 0.1278 | 0.616 | 500 MPR | 1/IF 0 | 500 | C_max_: 9.03.  C_max_ FE: 0.81.  AFE: 1.17.  AAFE: 1.30.  Within evaluation limits. Model C successful in validation with patient data (dataset C) |

Table S4. Summary table of all simulations conducted in this study with input values for various factors and the simulation outcome. Simulation number 1 was compared to in vivo dataset A (21), number 2-39 were compared to in vivo dataset B (16) and number 40 was compared to in vivo dataset C (30).
