# Supplementary material for: Using PBPK to Simulate Target Biopredictive Dissolution Profiles for Long‐Acting Injectables ‐ Where to Begin With Critical Bioavailability Attributes?
Source: CPT Pharmacometrics Syst Pharmacol. 2026 Feb 18;15(3):e70212. doi: 10.1002/psp4.70212 (PMC12916861; doi:10.1002/psp4.70212)
Supplement: Supplementary file 2 — Data S2: Supporting Information. [file PSP4-15-e70212-s001.docx]

Supplementary Information – In vivo datasets

| Time (hours) | Methylprednisolone plasma concentration (ng/ml) |
| --- | --- |
| 0.01 | 695.26 |
| 0.05 | 813.44 |
| 0.16 | 875.44 |
| 0.18 | 896.99 |
| 0.33 | 834.23 |
| 0.62 | 712.89 |
| 0.9 | 573.36 |
| 1.12 | 553.03 |
| 1.41 | 527.05 |
| 1.93 | 467.2 |
| 2.91 | 333.15 |
| 3.9 | 240.47 |
| 4.92 | 157.54 |
| 5.91 | 105.74 |
| 6.9 | 64.41 |
| 7.96 | 39.24 |
| 9 | 32.76 |
| 9.97 | 19.71 |

Table S2_1. In vivo dataset A used in step 1 to obtain disposition parameters for IM model (21).

| Time (hours) | Methylprednisolone plasma concentration (ng/ml) |
| --- | --- |
| 0 | 0 |
| 2 | 3.2 |
| 4 | 4.62 |
| 6 | 5.42 |
| 7 | 5.54 |
| 8 | 5.87 |
| 9 | 6.24 |
| 10 | 6.27 |
| 11 | 6.04 |
| 12 | 6.09 |
| 14 | 5.94 |
| 16 | 5.75 |
| 24 | 6.35 |
| 36 | 5.59 |
| 48 | 5.93 |
| 72 | 5.35 |
| 120 | 5.11 |
| 168 | 4.89 |
| 336 | 3.55 |
| 504 | 2.59 |
| 672 | 1.79 |
| 840 | 1.33 |
| 1008 | 0.96 |
| 1176 | 0.72 |

Table S2_2. In vivo dataset B used in steps 2-6 (16).

| Time (hours) | Methylprednisolone plasma concentration (ng/ml) | Error bars (+/-) |
| --- | --- | --- |
| 2.525697504 | 3.98056696 | 2.329276 |
| 5.991189427 | 5.715263014 | 3.456989 |
| 24.90455213 | 4.536853675 | 1.082889 |
| 74.00881057 | 5.166148805 | 4.813085 |
| 190.9544787 | 5.869072923 | 4.663799 |
| 354.4787078 | 3.123974915 | 2.769727 |
| 361.174743 | 7.34575545 | 4.754731 |
| 526.8135095 | 3.783182723 | 3.735216 |
| 690.3964758 | 6.161816512 | 5.479361 |
| 1026.490455 | 3.48217201 | 0.849065 |

Table S2_3. In vivo dataset C used in step 7 as a multidose external validation of IM model (30).
